# Splice-switching of the oncogenic *BCS1L* isoform suppresses ovarian cancer progression by disrupting mitochondrial function

**DOI:** 10.1038/s41419-026-08495-6

**Published:** 2026-03-03

**Authors:** Meining Xu, Zixiang Wang, Siyuan Yang, Gaoyuan Li, Xiyu Zhang, Ling Zhao, Lei Yang, Chunhong Qiu, Xianguang Feng, Kai Zhang, Bin Liu, Jian-jun Wei, Yuliang Li, Gang Liu, Baoxia Cui, Junchao Qin, Zhaojian Liu

**Affiliations:** 1 Key Laboratory of Experimental Teratology, Ministry of Education, Department of Obstetrics and Gynecology, Qilu Hospital, Department of Cell Biology, School of Basic Medical Science, Shandong University, Jinan, China; 2 Advanced Medical Research Institute, Shandong University, Jinan, China; 3 Department of Hepatobiliary Surgery, Shandong Provincial Third Hospital, Shandong University, Jinan, Shandong China; 4 Department of Interventional Medicine and Minimally Invasive Oncology, The Second Hospital of Shandong University, Jinan, China; 5 Department of Pathology, Northwestern University Feinberg School of Medicine, Chicago, IL USA; 6 Nephrology Research Institute of Shandong University, The Second Hospital of Shandong University, Jinan, China

**Keywords:** RNA metabolism, Gynaecological cancer, Cancer metabolism

## Abstract

Increasing evidences demonstrate that mitochondrial function is essential for cancer cell survival and metastasis. However, the role of mitochondrial metabolic reprogramming in ovarian cancer progression remains largely unknown. Here, we report that mitochondrial chaperone *BCS1L* generates two major alternative-spliced isoforms, a full-length isoform (*BCS1L-L*) and a short isoform lacking exon 2 (*BCS1L-S*). Interestingly, *BCS1L-L* is elevated in several human cancers, and it significantly increased oxidative phosphorylation and ATP production in the present work, which is required for the survival of cancer cells. In contrast, BCS1L-S was unable to localize to the mitochondria as BCS1L-L did, and this led to impaired metabolic function. Mechanistically, splicing factor *USP39* promoted exon 2 inclusion, thus facilitating the generation of oncogenic *BCS1L-L* and, thereby, maintaining mitochondrial homeostasis and survival of ovarian cancer cells. Importantly, we developed splice-switch antisense oligonucleotides (ASOs) that successfully induced exon 2 skipping and decreased *BCS1L-L* abundance, resulting in impaired tumor growth. These findings suggest that targeting oncogenic *BCS1L-L* by ASOs is a novel approach for ovarian cancer treatment.

## Introduction

Increasing evidences demonstrate that functional mitochondria are essential for cancer cells. Mitochondrial transfer from immune cells leads to tumor cell immune evasion [1]. Cancer cells can steal mitochondria from nerve cells to fuel their spread [2]. Mitochondrial oxidative phosphorylation (OXPHOS) and glycolysis cooperate to maintain the cellular energy balance, and cancer cells undergo significant metabolic reprogramming in order to support their rapid growth, proliferation, migration, and reshape the tumor microenvironment [3]. The Warburg effect is the most-studied metabolic change in which cancer cells favor glycolysis for energy production in the presence of sufficient oxygen [4]. The Warburg effect has been observed in various human cancers, including breast [5], colorectal [6], and lung cancer [7]. Reducing glycolytic energy production is mostly destined to fail as a therapeutic option [4], but emerging evidence has demonstrated that mitochondrial function is essential for cancer cells' viability, and this might be a viable therapeutic alternative [8].

Increasing evidence suggests that OXPHOS activity is upregulated in certain cancers and is associated with cancer drug resistance and metastasis [9–11]. For example, metastatic estrogen receptor-positive breast cancers are highly reliant on OXPHOS [12], and OXPHOS is upregulated in melanoma brain metastases while inhibition of OXPHOS in mouse models reduces metastases to the brain [13]. In addition, acute myeloid leukemia stem cells are reliant on OXPHOS for survival, and single-cell multiomics sequencing of ovarian cancer cells shows that both OXPHOS and glycolysis are increased [14]. It is clear, therefore, that metabolic plasticity enables cancer cells to switch between glycolysis and OXPHOS during tumorigenesis and metastasis.

OXPHOS is the process in which ATP is produced through the electron transport chain, which consists of four protein complexes (complexes I–IV) [15]. Among these complexes, complex III facilitates the transfer of electrons from ubiquinol to cytochrome *c*. Complex III consists of 11 subunits, with 10 subunits encoded by nuclear DNA and one (*MT-CYB*) encoded by mitochondrial DNA [16]. Mutations in genes encoding complex III subunits, including *MT-CYB*, *CYC1*, *UQCC2*, and *BCS1L*, have been shown to cause disease [17].

The *BCS1L* gene encodes the transmembrane chaperone, BCS1L, that is required for the assembly of mitochondrial respiratory chain complex III [18]. BCS1L contains a single transmembrane domain near the N-terminus spanning the mitochondrial inner membrane, an AAA (ATPases associated with diverse cellular activities) domain, and a mitochondrial targeting signal [19]. BCS1L facilitates the translocation and incorporation of a folded iron–sulfur protein into the core assembly of complex III [20]. Mutations in *BCS1L* cause GRACILE syndrome or Björnstad syndrome, both of which are associated with mitochondrial complex III deficiency and affect the brain, kidney, liver, heart, and skeletal muscles [21, 22]. In the current work, we demonstrated that *BCS1L* was alternatively spliced to yield two major isoforms, a full-length isoform (*BCS1L-L*) and a truncated isoform skipping exon 2 (*BCS1L-S*). We showed that, in contrast to BCS1L-L, BCS1L-S failed to localize to the mitochondria due to loss of the mitochondrial targeting signal. We also demonstrated that splicing factor *USP39* stimulates exon 2 inclusion to generate the oncogenic *BCS1L-L* isoform. Based on the *USP39*-binding region on *BCS1L*, we developed a splice-switching antisense oligonucleotide (ASO) that decreased *BCS1L-L* abundance and hence suppressed tumor growth. Our results suggest that targeting *BCS1L* with ASOs might be an effective approach for cancer therapy.

## Results

### Mitochondrial chaperone *BCS1L* is preferentially expressed in OXPHOS^high^ ovarian cancers

**Fig. 1:**
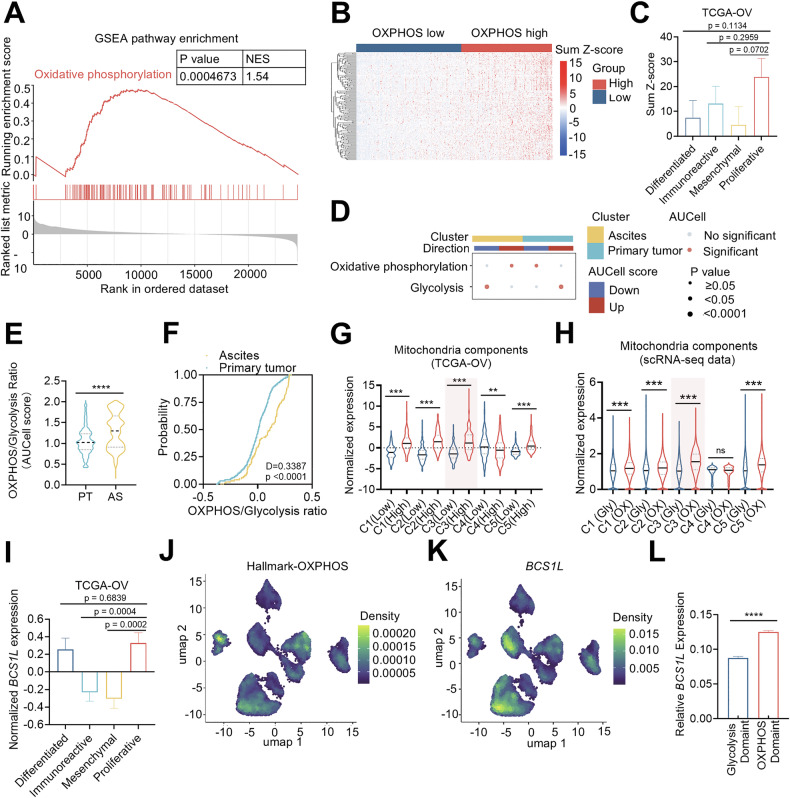
BCS1L is preferentially expressed in OXPHOShigh ovarian cancers. **A** GSEA pathway enrichment performed between ovarian cancer and normal tissues from GTEx-ovary (*n* = 180) and TCGA-OV (*n* = 374) datasets. Normalized enrichment score (NES) = 1.54, *p* = 0.0004673. **B** The heat map of 184 oxidative phosphorylation–associated genes expression in TCGA-OV (*n* = 374). The OXPHOS high (sum *Z*-score > 0, 175) and OXPHOS low (sum *Z*-score ≤ 0, 199) groups were distinguished in different colors. Each column is the summary of *Z*-score ranging from blue (−15) to red (15). **C** The sum score of oxidative phosphorylation–associated gene expression in cell subtypes from the TCGA-OV dataset, including differentiated subtype, immunoreactive subtype, mesenchymal subtype, and proliferative subtype. **D** Dot plot of enriched pathways of clusters originating from ascites or primary tumor. The dot size is proportional to the *p*-value, and the color indicates the significance of the AUCell score. **E** The ratio of OXPHOS to glycolysis evaluated by AUCell score in primary tumor and ascites in scRNA-seq. **F** The probability of primary tumor cells and ascites-derived cells mapping to different OXPHOS/glycolysis ratios from scRNA-seq. *D* = 0.3387, *p* < 0.0001. **G** Normalized expression of five mitochondrial complex main components from TCGA-OV, red shadow indicated the complex III. The cohorts (OXPHOS high and OXPHOS low) were split based on OXPHOS-associated genes expression. The complex main components were listed in Table S4. **H** Normalized expression of five mitochondrial complex components from scRNA-seq data, red shadow indicated the complex III. The cohorts (Glycolysis and OXPHOS) were split based on the AUCell score of OXPHOS and glycolysis. The complex main components were listed in Table S4. **I** The normalized *BCS1L* expression in cell subtypes from TGCA-OV, including differentiated subtype, immunoreactive subtype, mesenchymal subtype, and proliferative subtype. **J** UMAP plots of OXPHOS marker gene expression, the legend shows a color gradient of the normalized read count. **K** UMAP projection of *BCS1L* normalized expression, the legend shows a color gradient of the normalized read count. **L** The relative *BCS1L* expression in the OXPHOS-dominant subtype and glycolysis-dominant subtype was recognized in scRNA-seq. The two-tailed unpaired Student’s *t*-test was used to determine the p value in **E**–**H** and **L**, **p < 0.01, ***p < 0.001, ****p < 0.0001, ns: not significant. Data in **C**, **I**, and **L** are presented as means ± SEM.

To demonstrate the metabolic heterogeneity of ovarian cancer, we performed Gene Set Enrichment Analysis (GSEA) on The Cancer Genome Atlas-OV (TCGA-OV) and Genotype Tissue Expression-ovary (GTEx-ovary) databases, and found that the oxidative phosphorylation (OXPHOS) pathway was significantly enriched (Fig. 1A). Next, we classified OXPHOS^high^ and OXPHOS^low^ subgroups of 374 ovarian cancers from the TCGA-OV dataset based on 184 hallmark OXPHOS genes from MsigDB as previously proposed (Fig. 1B) [11]. Further analysis revealed that immunoreactive and proliferative subtypes of ovarian cancers exhibited higher OXPHOS score (Fig. 1C). Single-cell RNA sequencing (scRNA-seq) from the primary ovarian tumors and malignant ascites of 14 patients with advanced OC [23] showed that malignant ascites preferentially utilized OXPHOS rather than glycolysis compared to primary tumors (Fig. 1D). The OXPHOS/Glycolysis ratio was significantly higher in ascites than the primary tumors (Fig. 1E and F). We subsequently analyzed expression levels of core components of five mitochondrial complexes and observed that OXPHOS^high^ tumors exhibited higher expression of components of complex I–III, V, both in TCGA-OV and scRNA-seq data (Fig. 1G and H). Of note, complex III components had the highest expression level in OXPHOS^high^ tumors compared to OXPHOS^low^ tumors. We then focused on BCS1L, a chaperone responsible for the assembly of complex III. We analyzed *BCS1L* expression among four subtypes of ovarian cancer and observed that *BCS1L* exhibited higher expression levels in proliferative ovarian cancer subtypes in TCGA-OV data (Fig. 1I). Interestingly, scRNA-seq analysis revealed that *BCS1L* expression is closely associated with OXPHOS-dominant cancer cells (Figs. 1J, K and S1A). Moreover, *BCS1L* was preferentially expressed in OXPHOS-dominant subgroups compared to glycolysis-dominant subgroups (Fig. 1L). Collectively, these data indicate that ovarian cancer displays metabolic heterogeneity and that the expression of mitochondrial complex III subunit *BCS1L* is closely associated with OXPHOS^high^ tumors.

### The full-length isoform of *BCS1L* is the predominantly expressed isoform in ovarian cancer

**Fig. 2:**
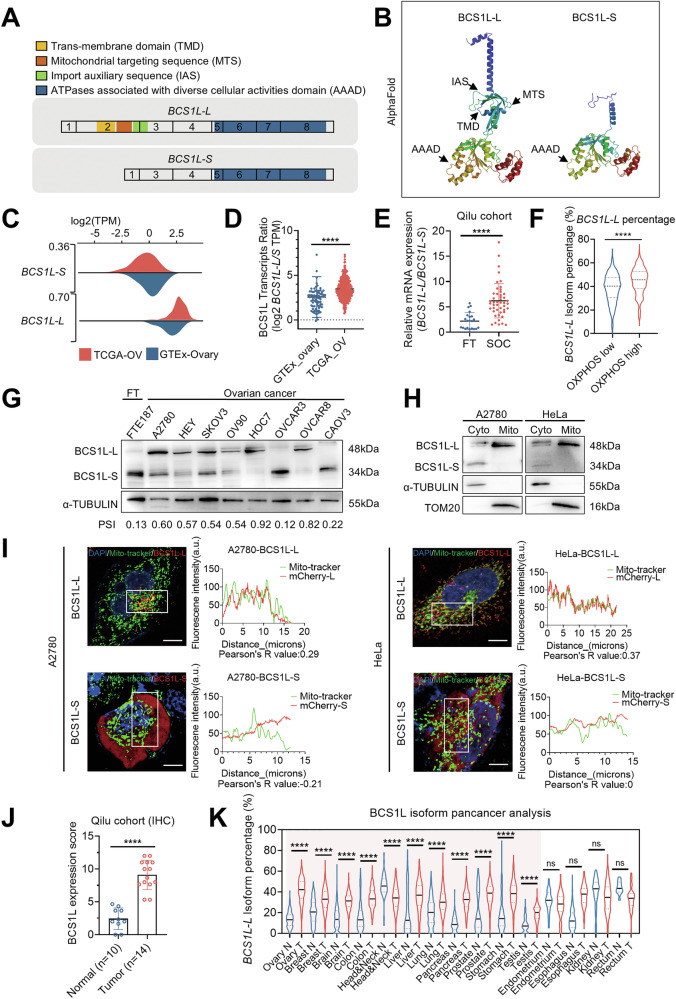
The full-length isoform of *BCS1L* is the predominantly expressed isoform in ovarian cancer. **A** Sequence analysis of the two prevalent *BCS1L* isoforms from the Ensemble database. BCS1L-L, ENST00000359273.7; BCS1L-S, ENST00000443791.5; TMD, trans-membrane domain; MTS, mitochondrial targeting sequence; IAS, import auxiliary sequence; AAAD, ATPases associated with diverse cellular activities domain. **B** The predicted structure of the two BCS1L isoforms by AlphaFold. TMD, trans-membrane domain; MTS, mitochondrial targeting sequence; IAS, import auxiliary sequence; and AAAD, ATPases associated with diverse cellular activities domain. **C** Isoform normalized expression (TPM) of *BCS1L-L* and *BCS1L-S* in SOCs and normal tissues according to the TCGA-OV (*n* = 374) and GTEx (*n* = 180) datasets. **D** The ratio of *BCS1L-L* to *BCS1L-S* was upregulated in SOCs (*n* = 374) compared with normal ovaries (*n* = 180). **E** The ratio of *BCS1L-L* to *BCS1L-S* was evaluated by qPCR in clinical samples (22 FT tissues and 47 SOC tissues). **F** The percentage of *BCS1L-L* isoform expression in the OXPHOS low or OXPHOS high group based on TCGA-OV. The classification of two metabolic groups was consistent with Fig. 1B. **G** Immunoblot analysis of BCS1L isoforms in a Fallopian tube epithelial (FTE187) and ovarian cancer cell lines. The upper band at 48 kDa indicated the long isoform and the lower band at 34 kDa indicated the short isoform. The PSI value was calculated using gray-scale analysis. **H** Immunoblotting analysis of BCS1L-L and BCS1L-S protein levels in A2780 and HeLa cells in the cytoplasm and mitochondria. α-TUBULIN and TOM20 were used as controls to indicate cytoplasmic and mitochondrial localization. **I** Confocal image of live A2780 and HeLa cells transfected with pUltra-hot-BCS1L-L and pUltra-hot-BCS1L-S plasmids for 72 h, showing the localization of BCS1L-L and BCS1L-S. Mito-tracker (green) indicated the mitochondrial localization, and DAPI (blue) was used to visualize the nuclei. Pearson’s *R* value was calculated by the ImageJ plug-in Color2. Scale bars, 10 µm. **J** The immunohistochemistry score of BCS1L expression levels in FT tissues (*n* = 10) and SOC tissues (*n* = 14) from the Qilu cohort. **K** Pan-cancer analysis of *BCS1L-L* isoform percentage according to the TCGA and GTEx datasets. The *p*-value (**D**–**F**, **J**, **K**) was obtained by an unpaired two-sided Student’s *t*-test. **p < 0.01, ***p < 0.001, ****p < 0.0001; ns: not significant.

To investigate whether *BCS1L* undergoes aberrant alternative splicing in cancer, we analyzed *BCS1L* splicing using the Ensemble database and found the *BCS1L* isoform switch was the most notable in TCGA-OV versus GTEx-ovary dataset among the other subunits, such as UQCRC1, UQCRC2, and CYC1 (Fig. S1B). According to the Ensemble database, the BCS1L transcript has 8 exons and 21 annotated splicing variants. Two prevalent isoforms, a full-length isoform (*BCS1L-L*) and an isoform lacking exon 2 (*BCS1L-S*), were characterized using the TCGA-OV and GTEx-ovary datasets (Fig. 2A). Protein structure prediction using AlphaFold showed that BCS1L-S lost the transmembrane domain, mitochondrial targeting sequence, and import auxiliary sequence that are found in BCS1L-L and are essential for mitochondrial localization (Fig. 2B). We next analyzed the expression of *BCS1L-L* (including exon 2) and *BCS1L-S* (skipping exon 2) using the TCGA-OV and GTEx-ovary datasets. *BCS1L-S* was predominantly expressed in normal tissues, whereas *BCS1L-L* was the dominant isoform in ovarian cancer tissues (Fig. 2C). Consistent with this, the *BCS1L-L*/*BCS1L-S* ratio was significantly increased in ovarian cancer tissues compared to normal tissues in the TCGA-OV and GTEx-ovary datasets (Fig. 2D). The increased *BCS1L-L*/*BCS1L-S* ratio in ovarian cancer was confirmed in our validation cohort by qPCR using isoform-specific primers (Fig. 2E). In two metabolic subtypes of ovarian cancer identified in Fig. 1B, the *BCS1L-L* percentage and the ratio of *BCS1L-L* to *BCS1L-S* were upregulated in the OXPHOS^high^ subtype of ovarian cancer (Figs. 2F and S1C). Furthermore, the expression of BCS1L-L and BCS1L-S was detected by immunoblot in different cell lines. Our data revealed that, unlike most of the ovarian cancer cell lines, *BCS1L-S* was the dominant isoform in OVCAR3 and CAOV3 cells (Fig. 2G), indicating these two cell lines have low OXPHOS and ATP production. We then analyzed OXPHOS-related genes from MsigDB using Cancer Cell Line Encyclopedia (CCLE) data and found that OVCAR3 and CAOV3 cells had a lower OXPHOS score compared to most of the ovarian cancer cell lines (Fig. S1D). Further immunoblot analysis of subcellular fractions indicated that BCS1L-S was enriched in the cytoplasmic and nuclear fractions, whereas BCS1L-L was enriched in mitochondria (Fig. 2H). We further determined the localization of BCS1L-L and BCS1L-S by overexpressing mCherry fusion proteins in A2780 and HeLa cells, and immunofluorescence analysis confirmed that BCS1L-S was unable to localize to the mitochondria like BCS1L-L (Figs. 2I and S1E, F). We next performed immunohistochemistry in 14 serous ovarian cancer (SOC) tissue samples (SOCs) and 10 fallopian tube (FTs) tissue samples and showed that the BCS1L protein was overexpressed in SOCs relative to FTs (Figs. 2J and S1G). Importantly, pan-cancer analysis showed that *BCS1L-L* is overexpressed in various cancer types, indicating its potential implications in cancer (Fig. 2K). These findings suggest that the full-length isoform is the dominant isoform in ovarian cancer.

### BCS1L-L contributes to the maintenance of mitochondrial homeostasis and the survival of ovarian cancer cells

**Fig. 3:**
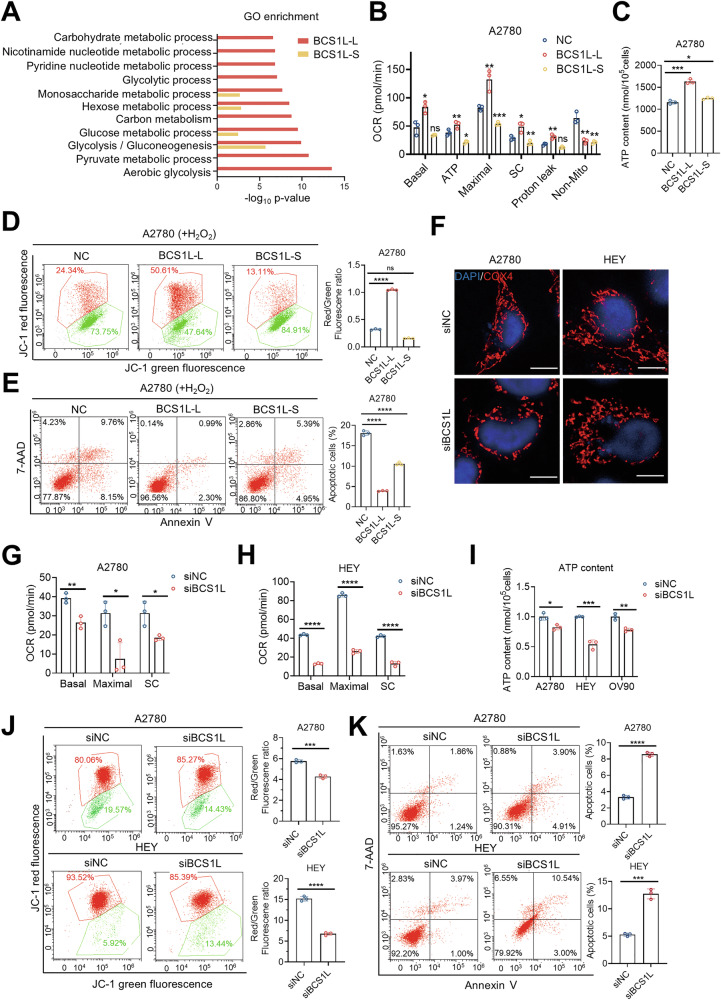
*BCS1L* contributes to the maintenance of mitochondrial homeostasis and the survival of ovarian cancer cells. **A** GO enrichment of proteins captured by the FLAG antibody from A2780 cells overexpressing BCS1L-L or BCS1L-S with FLAG tags. **B** Mitochondrial basal OCR, ATP production, Maximal OCR, Proton leak, non-mitochondrial respiration, and spare capacity (SC) were evaluated in A2780 cells with BCS1L-L or BCS1L-S overexpression for 72 h and control cells using an Agilent Seahorse XFe24. **C** ATP content per 10^5^ A2780 cells transfected with control, BCS1L-L, and BCS1L-S overexpression vectors for 72 h. **D** MMP was measured by JC-1 staining in A2780 cells with control and BCS1L-L or BCS1L-S overexpression for 72 h treated with 500 μM H_2_O_2_ for 4 h. Red fluorescence indicated normal MMP, while green fluorescence indicated abnormal MMP. **E** Apoptotic cells were analyzed by flow cytometry using Annexin V/7-AAD staining in A2780 cells with control and BCS1L-L or BCS1L-S overexpression for 48 h treated with 500 μM H_2_O_2_ for 4 h. Statistical analysis of apoptotic cells included early (Q2) and late (Q3) apoptotic cells. **F** Immunofluorescence of COX4 (red) in A2780 and HEY cells with BCS1L knockdown and control cells showing mitochondria morphology. DAPI was used to visualize the nuclei. Scale bars, 10 µm. **G** and **H** Mitochondrial basal OCR, maximal OCR, and SC (spare capacity) were evaluated in A2780 and HEY cells with BCS1L knockdown for 72 h using an Agilent Seahorse XFe96. **I** ATP content per 10^5^ cells in three ovarian cancer cell lines with BCS1L knockdown for 48 h (three independent experiments). **J** MMP was measured by JC-1 staining in A2780 and HEY cells with BCS1L knockdown for 72 h. Red fluorescence indicated normal MMP, while green fluorescence indicated abnormal MMP. **K** Apoptotic cells were analyzed by flow cytometry using Annexin V/7-AAD staining in A2780 and HEY cells with BCS1L knockdown for 72 h. Statistical analysis of apoptotic cells included early (Q3) and late (Q2) apoptotic cells. All results were repeated in three independent experiments and evaluated by a two-tailed unpaired Student’s *t*-test. *p < 0.05, **p < 0.01, ***p  < 0.001, ****p < 0.0001. Data are shown as the mean ± SD.

To investigate the functional role of BCS1L-L and BCS1L-S, we first characterized the interacting proteins of two BCS1L isoforms using immunoprecipitation coupled with mass spectrometry. Interestingly, GO enrichment analysis revealed that BCS1L-L, but not BCS1L-S, interacted with many proteins in mitochondrial metabolic pathways (Figs. 3A and S2A). We then measured respiratory capacity using a Seahorse XF96 analyzer in A2780 cells with BCS1L-L or BCS1L-S overexpression. Ectopic expression of BCS1L-L in A2780 cells resulted in higher basal OCR, maximal respiration, and mitochondrial respiratory capacity compared to BCS1L-S (Figs. 3B and S2B). Meanwhile, the ATP content was significantly increased in A2780 with BCS1L-L overexpression rather than BCS1L-S (Fig. 3C). Furthermore, JC-1 assays showed that BCS1L-L, but not BCS1L-S, increased the mitochondrial membrane potential (MMP) in A2780 cells treated with hydrogen peroxide (H_2_O_2_) (Fig. 3D). We further measured reactive oxygen species (ROS) levels and found BCS1L-L overexpression had a stronger capacity to clear ROS than BCS1L-S (Fig. S2C). We also measured apoptosis using Annexin V-PE/7-AAD staining and flow cytometry and found overexpression of BCS1L-L suppressed H_2_O_2_-induced ovarian cancer cell apoptosis, whereas BCS1L-S could not (Fig. 3E). Conversely, BCS1L knockdown in A2780 and HEY cells changed the mitochondrial morphology from a tubular to a fragmented state as evidenced by immunofluorescent staining of COX4 (Figs. 3F and S2D–F). Consistently, BCS1L knockdown significantly decreased basal and maximal OCR and ATP content in A2780 and HEY cells (Figs. 3G–I and S2G, H). Additionally, BCS1L knockdown generated a significant increase in ROS levels (Fig. S2I–L), and reduced MMP levels (Figs. 3J and S2M). Not surprisingly, knockdown of BCS1L suppressed cell proliferation (Fig. S2N), and induced significant spontaneous apoptosis in A2780, HEY, and OV90 cells (Figs. 3K and S2O). Taken together, these findings suggest that BCS1L-L is required for the maintenance of mitochondrial homeostasis and is important for ovarian cancer cell survival.

### The splicing switch between *BCS1L-L* and *BCS1L-S* is regulated by USP39 in ovarian cancer cells

**Fig. 4:**
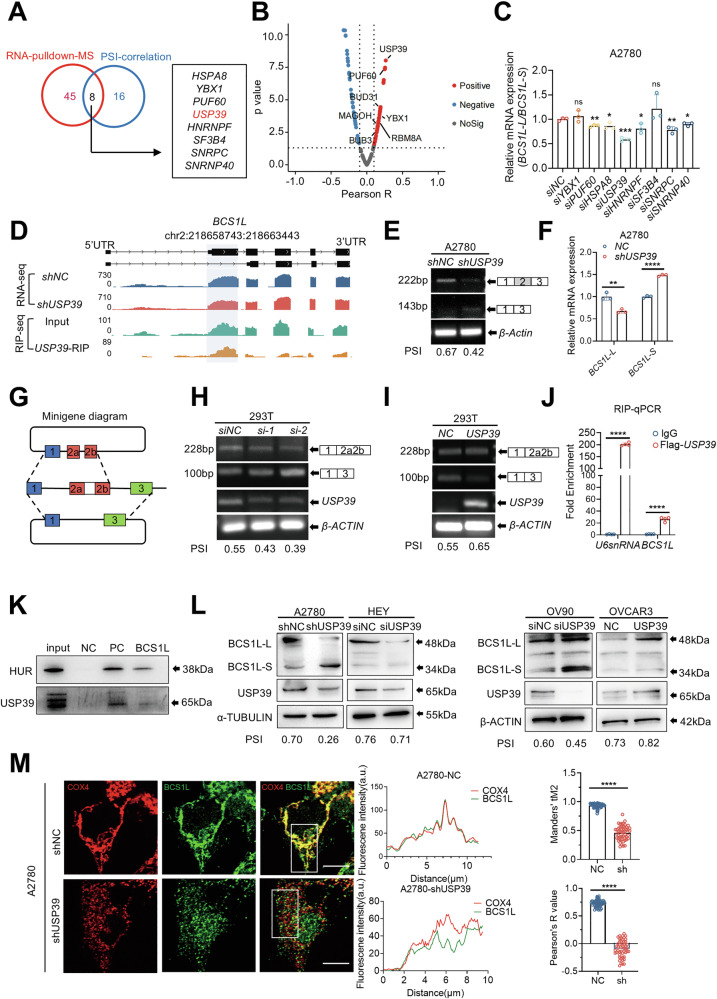
The splicing switch between *BCS1L-L* and *BCS1L-S* is regulated by USP39 in ovarian cancer cells. **A** Venn diagram of 53 *BCS1L*-bound splicing factors from the *BCS1L* RNA pulldown-MS in A2780 cells and 24 genes regulating *BCS1L* splicing derived from the TCGA-OV datasets (Pearson’s *R* > 0.15, *p* < 0.001). **B** Volcano plots of differentially expressed *BCS1L*-bound splicing factors between the TCGA-OV (*n* = 374) dataset and the normal tissue in GTEx-ovary (*n* = 180) datasets. |Pearson *R* | > 0.1 and *p*-value > 0.05 were considered significant. Positive correlations are shown in red, and negative correlations are shown in blue. **C** Relative *BCS1L-L/BCS1L-S* transcript expression was measured by qPCR in A2780 cells transfected with siRNAs targeting *YBX1*, *PUF60*, *HSPA8*, *USP39*, *HNRNPF*, *SF3B4*, *SNRPC*, and *SNRNP40* or a negative control for 48 h. **D** Sashimi plots of the alternative splicing pattern and USP39 direct binding sites in *BCS1L* were created with IGV using RNA-seq and RIP-seq data in A2780 cells. The light blue region indicates the alternative exon and the USP39-binding sites. **E** Semi-quantitative RT-PCR was performed to validate alternative splicing events in A2780 cells with USP39 knockdown using isoform-specific primers. **F** The relative expression ratio of *BCS1L-L/BCS1L-S* was analyzed in A2780 cells with USP39 depletion using isoform-specific primers. **G** Schematic diagram of the *BCS1L* minigene structure and alternative splicing products. **H** The expression of *BCS1L* minigene transcripts in 293T cells with USP39 depletion using different USP39 siRNAs as measured by semi-quantitative RT-PCR. **I** The expression of *BCS1L* minigene transcripts in 293T cells transfected with USP39 expression plasmids was measured by semi-quantitative RT-PCR. **J** The interaction between USP39 and *BCS1L* RNA was validated by RIP-qPCR of A2780 cells overexpressing USP39. U6snRNA served as the positive control. *n* = 3 biologically independent experiments. **K** RNA pull-down assay showing the interaction between the *BCS1L* RNA and USP39 protein in A2780 cells. Androgen receptor 3´-UTR RNA was used as the positive control and poly (A)_25_ RNA was used as the negative control. **L** The protein expression of BCS1L-L and BCS1L-S was determined by western blot in A2780, HEY, and OV90 cells with USP39 knockdown and in OVCAR3 cells with USP39 overexpression. The top band at 48 kDa indicates BCS1L-L, and the bottom band at 34 kDa indicates BCS1L-S. **M** Representative confocal images of control A2780 cells and A2780 cells with USP39 knockdown showing co-localization of BCS1L (green) and COX4 (red). DAPI was used to visualize nuclei. Scale bars, 10 µm. Images are representative of at least three independent experiments. Co-localization coefficients, including Pearson correlation coefficient and Mander’s coefficient were quantified by Image J (*n* = 50 cells for control, *n* = 50 cells for USP39 knockdown). The *p*-value (**C**, **F**, **J**, **M**) was obtained by Student’s unpaired *t*-test and and the results are presented as the mean ± SD. *p < 0.05, **p < 0.01, ***p < 0.001, ****p < 0.0001.

We next sought to identify the splicing factors that regulate the alternative splicing of the *BCS1L* gene. We conducted an RNA pull-down assay followed by mass spectrometry in A2780 cells using an in vitro-transcribed biotin-labeled RNA probe, and mass spectrometry data identified 53 RNA-binding proteins that could potentially bind to *BCS1L* pre-mRNA (Fig. 4A). Furthermore, 24 of 134 core splicing factors showed co-expression correlation with *BCS1L* based on the TCGA-OV RNA-seq data (Fig. 4B). Overlap analysis of these three data sets showed that *HSPA8*, *YBX1*, *PUF60*, *USP39* and *HNRNPF* were the top five splicing factors putatively interacting with *BCS1L* (Fig. 4A). We subsequently conducted an RNAi screen targeting these splicing factors and measured the *BCS1L-L*/*BCS1L-S* ratio using isoform-specific primers. We found that *USP39* had the greatest impact on the splicing switch of *BCS1L* (Figs. 4C and S3A).

As a spliceosome component, *USP39* has been shown to be highly expressed in various human cancers [24–26]. To determine whether *USP39* regulates the splicing switch of *BCS1L* directly, we analyzed splicing events using RNA-seq data in A2780 cells upon USP39 knockdown. We found that downregulation of *USP39* led to the skipping of exon 2 in *BCS1L*, thus promoting the formation of *BCS1L-S*. Consistent with this, RIP-seq showed that *USP39* binds to exon 2 of *BCS1L* (Fig. 4D). We validated the splicing switch of *BCS1L* using semi-quantitative RT-PCR and found that downregulation of *USP39* generated more *BCS1L-S* and less *BCS1L-L* (Fig. 4E). We further performed qPCR and observed a significant decrease in the *BCS1L-L*/*BCS1L-S* ratio in A2780 cells upon USP39 knockdown (Fig. 4F). A *BCS1L* minigene spanning alternative exons or introns close to the exon-intron junction was constructed and transfected into 293T cells with USP39 depletion or overexpression (Fig. 4G). The minigene reporter showed that depletion of USP39 promoted exon 2 skipping and resulted in *BCS1L-S* (Fig. 4H). In contrast, ectopic expression of USP39 retained exon 2 to generate more *BCS1L-L* (Fig. 4I). To obtain further evidence for the direct regulation of *USP39* on the splicing switch of *BCS1L*, an RIP assay was performed using an anti-FLAG antibody in A2780 cells. RIP-QPCR showed that USP39 bound to exon 2 of *BCS1L* pre-mRNA (Fig. 4J). Additionally, we conducted an RNA pull-down assay to further confirm that USP39 was pulled down by the biotin-labeled *BCS1L* pre-mRNA, indicating the direct interaction between USP39 and *BCS1L* pre-mRNA (Fig. 4K). The protein level of BCS1L-L was significantly decreased while the BCS1L-S level was increased upon USP39 knockdown in A2780, HEY, and OV90 cells, as evidenced by immunoblotting, whereas overexpression of USP39 in OVCAR3 cells had the opposite effects (Fig. 4L). Consistent with these results, co-immunofluorescence staining of BCS1L-L and COX4 showed that loss of USP39 resulted in fragmented mitochondria, reduced BCS1L-L protein levels, and reduced BCS1L-L localization in the mitochondria (Fig. 4M). Collectively, these findings strongly suggest that the *BCS1L-L*/*BCS1L-S* splicing switch in ovarian cancer cells is regulated by USP39.

### The *USP39-BCS1L* axis mediates mitochondrial OXPHOS and suggests a therapeutic vulnerability for ovarian cancer

**Fig. 5:**
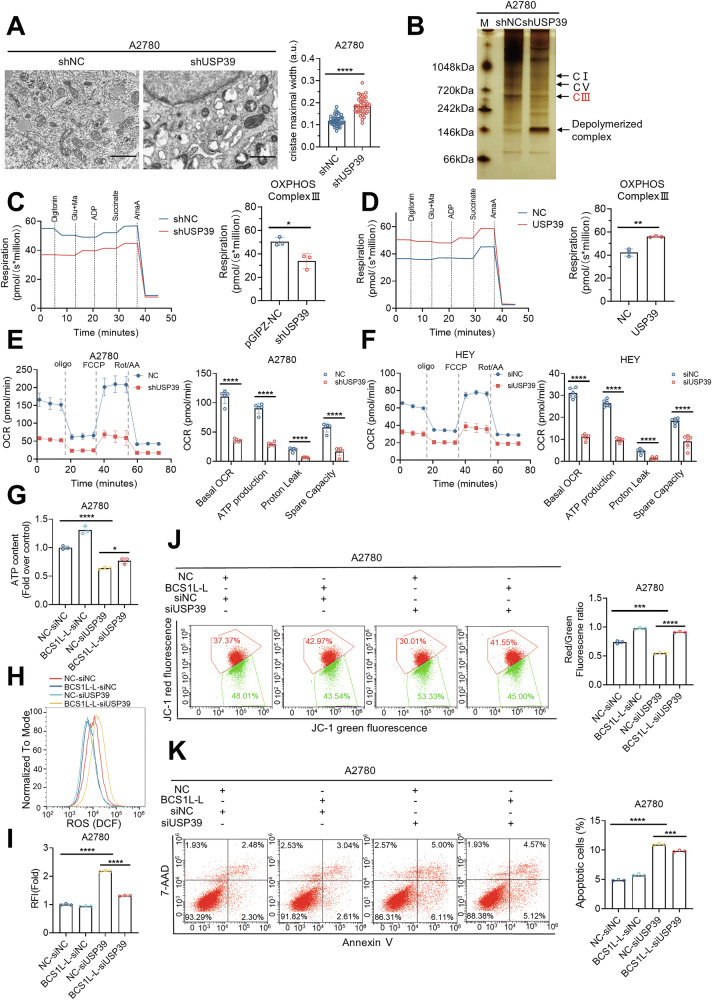
The *USP39-BCS1L* axis mediates mitochondrial OXPHOS and suggests a therapeutic vulnerability for ovarian cancer. **A** Transmission electron microscopy in control and A2780 cells with USP39 knockdown. Scale bars, 2 µm. Morphometric analysis of mitochondrial cristae was performed in a blinded fashion on at least five mitochondria per cell from six randomly selected cells, and maximal cristae width was measured using Image J. **B** Silver staining of BN-PAGE indicated mitochondrial OXPHOS assembly in control and USP39 knockdown A2780 cells. **C** and **D** Electron transport chain Complex III activities in control and USP39 knockdown A2780 cells were evaluated by substrate-uncoupler-inhibitor-titration (SUIT) on a high-resolution Oxygraph-2k respirometer. Complex activities were normalized to that of CS (*n* = 3). Quantification of OCR measurements in A2780 cells (**E**) and HEY cells (**F**) after USP39 knockdown with the pharmacological inhibitors of metabolism (left). Individual parameters of mitochondrial function, including basal respiration, ATP production, proton leak, and spare respiratory capacity, were calculated according to the manufacturer’s protocol with non-mitochondrial oxygen consumption adjustment (right). Rot/AA, rotenone/antimycin A. **G** Quantification of ATP levels in A2780 cells for investigating the potential of BCS1L to rescue the loss of USP39. **H** and **I** ROS distribution was measured by flow cytometry in A2780 cells transfected with siUSP39 and BCS1L overexpression vector for 48 h. **J** Measurements and quantification of MMP (by JC-1) in A2780 cells with USP39 knockdown and BCS1L overexpression for 72 h. **K** Apoptotic A2780 cells after USP39 depletion together with BCS1L overexpression for 48 h were analyzed by flow cytometry using Annexin V/7-AAD staining. Three biological replicates were conducted in all functional experiments, and the *p*-value was calculated by two-tailed Student’s unpaired *t*-test (**A**, **C–G**, **I–K**), *p < 0.05, **p < 0.01, ***p < 0.001, ****p < 0.0001; ns: not significant. Data are shown as the mean ± SD.

To investigate the involvement of the *USP39-BCS1L* axis in mitochondrial OXPHOS in ovarian cancer cells, we established A2780 cell lines with stable USP39 knockdown. We performed RNA-seq on USP39 knockdown and control cells and identified 868 upregulated genes and 476 downregulated genes (Fig. S3B). Gene Ontology (GO) analysis showed that *USP39* target genes were enriched in mitochondrial-related biological processes, including complex assembly, mitochondrial gene expression, and ATP synthesis coupled with electron transport (Fig. S3C). We next assessed mitochondrial morphology and function in ovarian cancer cells with USP39 knockdown or overexpression (Fig. S3D). Electron tomography showed that the mitochondria were fragmented and mitochondrial cristae were decreased in USP39 knockdown A2780 cells (Fig. 5A). Mitochondrial BN-PAGE showed decreased complex III levels, with minimal changes in other complexes (Fig. 5B). Next, mitochondrial respiration was measured using Oroboros 2K oxygraphy respirometry and showed decreased OXPHOS activity, reduced levels of the electron transfer system, and severely impaired complex III activity in USP39 knockdown A2780 cells, whereas overexpression of USP39 in A2780 cells had the opposite effects (Fig. 5C and D). Meanwhile, the basal OCR, ATP production, and spare respiratory capacity were significantly decreased in A2780 and HEY cells after USP39 knockdown (Figs. 5E, F and S3E). We next evaluated ROS level and found that loss of USP39 led to an increase in ROS production in A2780 and HEY cells, whereas overexpression of USP39 suppressed ROS levels in A2780 cells (Fig. S3F). Additionally, the mitochondrial membrane potential (MMP) was decreased upon USP39 depletion, while overexpression of USP39 had the opposite effect (Fig. S3G).

We next characterized the functional interplay between *USP39* and *BCS1L* in the maintenance of mitochondrial homeostasis and survival of ovarian cancer cells. We first conducted rescue experiments in A2780 cells and found that BCS1L-L overexpression restored the reduced ATP production induced by USP39 knockdown (Figs. 5G and S3H). We also measured ROS and MMP levels and found that forced expression of BCS1L-L could attenuate the increased ROS and decreased MMP levels caused by USP39 knockdown (Fig. 5H–J). Moreover, USP39 knockdown-induced apoptosis was effectively attenuated by BCS1L-L overexpression in A2780 cells as measured by Annexin V-PE/7-AAD staining and flow cytometry (Fig. 5K). These findings suggest that *USP39* mediates mitochondrial OXPHOS and the survival of ovarian cancer cells partially through regulating the alternative splicing of *BCS1L* to generate *BCS1L-L*.

### Splice-switching ASOs targeting *BCS1L* exert antitumor effects in ovarian cancer

**Fig. 6:**
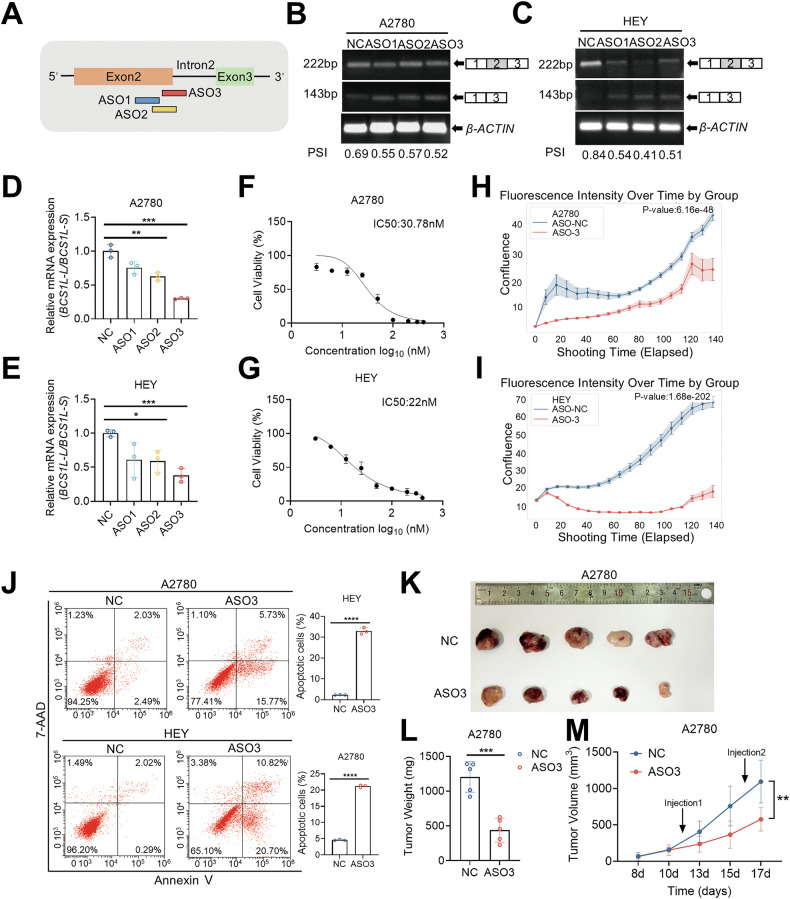
Splice-switching ASOs targeting *BCS1L* exert antitumor effects in ovarian cancer. **A** The schematic diagram for designing ASOs targeting the alternative splicing of *BCS1L*. **B** and **C** The inclusion of *BCS1L* exon 2 in response to three ASOs was analyzed by RT-PCR in A2780 cells (200 nM ASOs) and HEY cells (100 nM ASOs) for 48 h. **D** and **E** Isoform-specific primers were used in qPCR to determine the ratio of *BCS1L-L* to *BCS1L-S* in A2780 and HEY cells after treatment with the three ASOs. **F** and **G** The IC_50_ of ASO3 after treatment for 120 h was calculated in A2780 cells (30.78 nM ASO3) and HEY cells (22 nM ASO3). **H** and **I** The cell proliferation assay was performed using an Incucyte S3 live-cell imaging and analysis system in A2780 and HEY cells treated with 200 nM ASO3 and 80 nM ASO3, respectively, compared to corresponding controls (*n* = 3) for five days. Cell proliferation was compared to the initial state (time = 1 day). The shaded areas indicate the mean ± SEM. The *p*-value was calculated by two-way ANOVA. **J** Apoptotic cells stained with Annexin V-PE/7-AAD were detected by flow cytometry after ASO3 treatment (400 nM ASO3 for A2780 cells and 200 nM ASO3 for HEY cells) for 48 h. The percentage of apoptotic cells, including both early and late apoptotic cells. Three biological replicates were conducted in all functional experiments. **K** ASO3 and ASO-control in PBS were injected intratumorally into subcutaneous tumor xenografts using A2780 cells (*n* = 5 NOD-SCID mice per group). The arrow indicates the injection time. The tumor weight (**L**) and volume (**M**) were measured for each group. The p-values were determined by two-tailed unpaired Student’s *t*-test (**D**, **E**, **J**, **L**, **M**), *p < 0.05, **p < 0.01, ***p < 0.001, ****p < 0.0001; ns: not significant. Data are shown as the mean ± SD.

Splice-switching ASO therapies have been successfully developed to treat genetic diseases such as spinal muscular atrophy [27]. In order to develop splice-switching ASOs targeting *BCS1L*, we designed ASOs flanking the exon-intron boundaries of exon 2 near the USP39 binding site (Fig. 6A). Three ASOs were transfected into A2780 and HEY cells for 48 h, and splice-switching of *BCS1L* was examined using RT-PCR. All three ASOs could decrease *BCS1L-L* and increase *BCS1L-S* levels in A2780 and HEY cells (Fig. 6B and C), but qPCR using isoform-specific primers showed that ASO3 led to a greater *BCS1L-S/BCS1L-L* ratio compared to the other two ASOs in A2780 and HEY cells (Fig. 6D and E) in corroboration with protein expression (Fig. S4A), and was therefore used for subsequent experiments. The half-maximal inhibitory concentration (IC_50_) value for ASO3 was 30.78 nM in A2780 cells (Fig. 6F) and 22 nM in HEY cells (Fig. 6G), whereas it dropped to 945 nM in Normal Human Dermal Fibroblasts (NHDF) (Fig. S4B). We subsequently examined the inhibitory effect of ASO3 on A2780 and HEY cells using IncuCyte Live Cell Imaging experiments and found that ASO3 significantly inhibited A2780 and HEY cell proliferation (Fig. 6H and I). We further measured mitochondrial respiration in A2780 and HEY cells treated with ASO3 using a Seahorse XF Analyzer, and we observed a significant decrease in basal respiration and ATP production in both cell lines upon ASO3 treatment (Fig. S4C–F). Meanwhile, a significant increase in ROS levels was observed in A2780 and HEY cells in response to ASO3 treatment (Fig. S4G and H). We next performed Annexin V-PE/7-AAD staining followed by flow cytometry to measure apoptosis after ASO3 treatment and found that the proportion of Annexin-V-positive cells significantly increased in ASO3-treated A2780 and HEY cells compared to control cells (Fig. 6J), indicating that ASO3 induced apoptosis in the two tested cell lines. Moreover, the activity of mitochondrial respiratory complex III was significantly decreased after ASO3 treatment (Fig. S4I). In xenograft models using A2780 cells, intratumoral injection of ASO3 suppressed tumor growth and reduced tumor burden (Fig. 6K–M). These results suggest that targeting *BCS1L* splice switching with ASOs has significant antitumor effects in ovarian cancer.

## Discussion

Growing evidence suggests that mis-splicing plays an important role in the occurrence and development of many types of cancer [28], and targeting altered splicing has been shown to be a potential treatment option for cancer [29]. In this study, we characterized two most abundant isoforms of the *BCS1L* gene: a full-length isoform and a truncated isoform lacking exon 2. Our results showed that BCS1L-L is enriched in mitochondria and is required for the maintenance of mitochondrial homeostasis. In contrast, BCS1L-S failed to localize to the mitochondria, leading to impaired mitochondrial function. Interestingly, *BCS1L-L* was found to be markedly upregulated in various cancer types compared with normal tissues. Consistent with this, we found that *BCS1L-L* contributed to the maintenance of mitochondrial homeostasis and the survival of ovarian cancer cells, thus suggesting that *BCS1L-L* is a novel oncogenic driver and a potential therapeutic target in ovarian cancer.

We provide strong evidence that splice isoform switching of *BCS1L* is mediated by the splicing factor *USP39*. *USP39* has been shown to be a regulator of splicing fidelity, and its depletion produces proteotoxic splice variants [30]. In addition, *USP39* is required for the efficient splicing of *CTLA4* in tumor-infiltrating Treg cells [31], and our previous work showed that U*SP39* facilitates the efficient splicing of *HMGA2* and thereby increases the malignancy of ovarian cancer cells [26]. Here, we performed RNA pull-down followed by mass spectrometry to identify splicing factors that directly bind and regulate the alternative splicing of *BCS1L*. Among RNA-binding proteins that bind to *BCS1L* pre-mRNA (Fig. 4A), USP39 regulates the alternative splicing of *BCS1L* and increases the level of *BCS1L-L*. We also performed RIP-seq to verify the interaction between the USP39 protein and *BCS1L* pre-mRNA (Fig. 4D). Importantly, USP39 showed co-expression with the *BCS1L* gene based on TCGA-OV RNA-seq data. Thus, our data demonstrate that *USP39* directly regulates the alternative splicing of *BCS1L*.

Targeting OXPHOS has great potential for anti-tumor treatment, and several novel compounds that target OXPHOS and the electron transport chain are currently being developed. For example, atovaquone is a mitochondrial complex III inhibitor that exhibits antitumor effects on acute myeloid leukemia and gynecological cancers [32]. Atovaquone has entered phase II clinical trial for the treatment of platinum-resistant ovarian cancer (NCT05998135). In addition, targeting mitochondrial complex I has been shown to overcome chemoresistance in high-OXPHOS pancreatic cancer cells [33], and IACS-010759 has been shown to increase the efficacy of immunotherapy in preclinical studies [34]. However, Atovaquone and IACS-010759 are small molecules that target OXPHOS in mitochondria; the subcellular delivery barriers and low specificity limit their clinical potential. In this study, we developed splice-switching ASOs targeting *BCS1L* that could reduce *BCS1L-L* levels, thereby suppressing OXPHOS functions and inhibiting tumor growth. ASOs targeting *BCS1L-L* have high specificity and safety because *BCS1L-L* is the dominant isoform in ovarian cancer tissues but not in normal tissues. ASOs offer a promising strategy for the treatment of various diseases, including genetic disorders and cancer. A total of 12 ASO drug products have been commercially approved by the US Food and Drug Administration (FDA) [35]. Nevertheless, efficient delivery into target cells remains a major obstacle, which limits the broad application of ASOs. The lipid nanoparticle (LNP) and antibody-oligonucleotide conjugates (AOCs) present a promising solution to overcome the delivery challenge associated with ASOs [36]. Our findings thus suggest that ASO-mediated *BCS1L* exon 2 skipping is a promising strategy for cancer therapy [37].

In summary, we show that *BCS1L* produces two alternatively spliced isoforms with distinct cellular localization and function. Unlike BCS1L-L, BCS1L-S failed to localize to the mitochondria and thus led to impaired mitochondrial function. Our data further revealed that splicing factor *USP39* promotes the alternative splicing of *BCS1L* to generate the oncogenic isoform *BCS1L-L*, thereby maintaining mitochondrial homeostasis and supporting the survival of ovarian cancer cells. Our results also provide evidence that targeting *BCS1L* splice switching with ASOs significantly decreases *BCS1L-L* and exerts antitumor effects in ovarian cancer. The *USP39*-*BCS1L* axis thus appears to mediate mitochondrial OXPHOS, and future therapies might be developed that exploit this therapeutic vulnerability of ovarian cancer.

## Materials and methods

### Cell lines and cell culture

A2780, HEY, FTE187, and HEK293T cells were obtained from the Jian-Jun Wei lab at Northwestern University. Human ovarian cancer cell lines OV90, SKOV3, OVCAR3, OVCAR8, and CAOV3 were purchased from the American Type Culture Collection. A2780, HEY, OV90, HeLa, CAOV3, and HEK293T cells were cultured in high-glucose Dulbecco’s modified Eagle’s medium (DMEM) (Macgene). SKOV3 cells were cultured in McCoy’s 5A medium. FTE187 cells were cultured in medium consisting of a 1:1 mixture of Medium 199 and MCDB105 medium (Sigma-Aldrich). OVCAR8 and OVCAR3 cells were cultured in RPMI 1640 (Macgene). All media were supplemented with 10% fetal bovine serum (Gibco) and 1% penicillin/streptomycin (Macgene), and all cell lines were validated by Short Tandem Repeat (STR) profiling and mycoplasma testing.

### Mice

We obtained female non-obese diabetic/severe combined immunodeficiency disease (NOD/SCID) mice (6–8 weeks old) from Beijing Vital River Laboratory Animal Technology. In the subcutaneous xenograft model, an equal number (1 × 10^6^) of A2780 cells was harvested and resuspended in 0.1 mL PBS for subcutaneous injection. Female NOD/SCID mice were randomly divided into two groups (5 mice per group) and were housed in a cage at 20–25 °C and 50% humidity with a 12 h light/dark cycle. After the subcutaneous tumor reached 5 mm in diameter, 5 nmol ASO (Tsingke) was intratumorally injected twice. The mice were anesthetized on day 17 after the injection, and the tumor volume and weight were measured. Tumor volume was calculated with the following formula: tumor volume (mm^3^) = *a* × *b*^2^ × *π*/6, where ‘*a*’ is the longest diameter and ‘*b*’ is the shortest diameter. The animal experiment was granted by the Ethics Committee of Shandong University (SDULCLL2019-2-08).

### Human tissue samples

All evaluated FT and SOC tissues were obtained from 2009 to 2015 at Qilu Hospital. The SOC specimens were obtained from patients who were diagnosed with primary ovarian cancer and who had undergone no previous surgeries or chemotherapies. The FT tissues were collected from patients undergoing total hysterectomy and bilateral salpingo-oophorectomy for uterine diseases or benign neoplastic adnexal pathological changes. All patients provided informed consent. Immediately after tumor excision, patient samples were sliced into 5 mm^3^ cubes and were divided into two parts. One part was immersed in 10 volumes of RNALater for RNA extraction, and the other part was fixed in formol for histopathology investigations.

### Plasmid construction and cell transfection

The pENTER overexpression plasmid was from WZ Biosciences. cDNA containing a full-length open-reading frame of BCS1L-L was amplified using the BCS1L-pENTER plasmid as the template with the ClonExpress II One Step Cloning Kit (Vazyme), and *BCS1L* transcript variant 1 (NM_004328) was amplified from position 430 to the stop codon to produce recombinant BCS1L-S lacking the N-terminal transmembrane domain. The PCR products were, respectively, cloned into pUltra-hot-BSD (Addgene) and pCDH-Spytag-3xFLAG (Genechem) for the expression of BCS1L-L-mCherry fusion protein and BCS1L-L-3xFLAG fusion protein. The USP39 sequence was extracted from HA-USP39 (Addgene) using the EcoR1 and Xho1 restriction sites and was ligated into PCMV using the ClonExpress II One Step Cloning Kit (Vazyme, C112-02). USP39 small interfering RNA (siRNA) was synthesized by RiboBio, and the ASOs were synthesized with phosphorothioate modifications to the nucleobases in order to enhance the pharmacologic properties of the ASOs. Plasmids, siRNAs, and ASOs were transfected into cells using jetPRIME transfection reagent (PolyPlus) following the manufacturer’s instructions. RNA and protein were extracted 48 or 72 h after transfection. The siRNA and ASO sequences used in this study are listed in the Supplementary Table 3.

### Lentiviral constructions and transductions

The pGIPZ-shUSP39, PCMV-USP39, pCDH-Spy-3xFLAG-BCS1L, and pUltra-hot-BCS1L plasmids were designed and cloned previously, and the lentivirus vectors were transfected into HEK293T cells together with psPAX2 and pMD2.G to produce lentivirus particles. Stable cell lines were established by lentivirus infection followed by puromycin (2 μg/ml) selection for 2 weeks.

### Seahorse assay

The mitochondrial respiratory capacity was determined using an XF Cell Mito Stress Test Kit (Agilent Technologies). For whole-cell studies, cells were seeded in the XFe96 plates at a density of 1 × 10^4^ cells per well or in the XFe24 plates at a density of 1 × 10^5^ cells and were incubated for 24 h at 37 °C. The next day, the cells were incubated with the base medium containing 2 mM L-glutamine, 1 mM sodium pyruvate, and 10 mM glucose for 1 h prior to the assay. The cells were treated sequentially with 1.5 μM oligomycin, 1 μM FCCP (p-trifluoromethoxy carbonyl cyanide phenylhydrazone, a reversible inhibitor) (2 μM FCCP for BCS1L-L and BCS1L-S overexpression), and 0.5 μM rotenone/antimycin A (a mitochondrial complex I inhibitor and a mitochondrial complex III inhibitor, respectively). Data were analyzed by XF Stress Test Report Generators (Agilent Technologies) and were expressed as pmols/min for OCR and mpH/min for ECAR, respectively. Mitochondrial basal OCR was quantified using a Seahorse Mito Stress Test kit, maximal OCR was calculated as the OCR after FCCP minus the OCR after antimycin, and the spare respiratory capacity was calculated as ((OCR following FCCP)–baseline OCR)/baseline OCR.

### Mitochondrial orotate production

A2780 cells were trypsinized and washed three times with PBS, re-suspended in ice-cold Mir05 medium (0.5 mM EGTA, 3 mM MgCl_2_, 60 mM K-lactobionate, 20 mM taurine, 10 mM KH_2_PO_4_, 110 mM sucrose, 1 g/L essential fatty acid-free bovine serum albumin, and 20 mM HEPES, pH 7.1 at 30 °C), and transferred to the chamber of an Oxygraph-2k instrument (Oroboros). The cells were permeabilized with 5 μg digitonin per 10^6^ cells, and the respiratory rates were recorded at 37 °C in 2 mL glass chambers. Leak respiration was measured in the presence of the NADH-linked substrates glutamate (10 mM) and malate (2 mM), and activation of ATP synthesis was induced by the addition of 1.5 mM ADP. Next, succinate (10 mM) was added to fuel electrons via complex II, thus reconstituting the TCA cycle function and inducing convergent NADH- and succinate-linked electron flow through CI and CII into the Q-junction. Inhibition of complex III by antimycin A (2.5 µM) injection revealed the complex III respiratory capacity.

### MMP and ATP measurement

ATP levels were assessed by an enhanced ATP Assay Kit (Beyotime, S0027) according to the manufacturer’s protocol. MMP and ROS production were assessed by flow cytometry using the fluorescent probes JC-1 (MultiSciences Biotech,70-MJ101) and DCFH-DA (Beyotime, S0027). An equal number of cells were seeded in six-well plates, and after culturing for 48 h, the cells were digested with ethylenediaminetetraacetic acid (EDTA)-free trypsin (Macgene, CC035) for 3 min, washed once with PBS, and stained with fluorescent dye in Hank’s balanced salt solution for 20 min at 37 °C in the dark. After three washes with PBS, the samples were immediately analyzed by CytoFLEX S (Beckman Coulter Life Sciences). FlowJo (version 10.8.1) was used to analyze the flow cytometry data.

### Apoptosis analysis

An Annexin V-FITC Apoptosis Detection Kit (Vazyme, A213-01) was used to measure the rate of both early and late-stage apoptotic cells. A2780 and HEY cells in six-well plates were collected and incubated with Annexin V-PE and 7-AAD. A total of 1 × 10^4^ cells were analyzed within 20 min by CytoFLEX S (Beckman Coulter Life Sciences). USP39 and BCS1L overexpressing cell lines were treated with H_2_O_2_ (500 nM) for 4 h before detecting cell apoptosis.

### Cell proliferation assays

Both a methyl-thiazolyl diphenyl-tetrazolium bromide (MTT) assay and Incucyte S3 live-cell analysis system were used to determine cell viability and proliferation. Briefly, A2780 and HEY cells were seeded in 96-well plates at densities of 2 × 10^3^ and 1 × 10^3^ cells per well. MTT solution (5 mg/ml) was incubated with the cells for 4 h at 37 °C. The absorbance of the MTT-formazan product was measured using a microplate reader at 570 nm. Cell growth was monitored over the following 5 days, and the IC_50_ was determined 48 h after treatment. According to the manufacturer’s instructions, cells seeded in 96-well plates were imaged every 8 h in the IncuCyte Zoom HD/2CLR time-lapse microscopy system (Sartorius) equipped with an IncuCyte Zoom 10× Plan Fluor objective (Sartorius). Imaging was performed for 5 days at 37 °C. Analysis parameters for basic analyzer (endpoint and confluence) processing definitions were optimized individually for each cell type. The optimized processing definitions were subsequently used for real-time image analysis.

### RNA isolation and PCR analysis

Total RNA from cells was extracted with a Cell Total RNA Isolation Kit (Foregene) following the manufacturer’s protocol. Total RNA from human samples, including SOCs and FTs, was extracted with a Total DNA/RNA/Protein Isolation Kit (OMEGA Bio-tek). RNA was reverse transcribed into cDNA using the HiScript II Q RT SuperMix for qPCR Kit (Vazyme). The resulting cDNAs were quantified by real-time QPCR using the SYBR Green mix (Vazyme) on an Applied Biosystems QuantStudio 3. Tubulin served as the endogenous control. The relative mRNA levels were calculated using the ΔΔCt method. Semi-quantitative RT-PCR was used to analyze alternative splicing products. Primer sequences were designed for the constitutively expressed flanking exons, and 2× Taq Master Mix (Dye Plus) (Vazyme, P112-01) was used to simultaneously amplify isoforms that included or skipped the target exon. The primer sequences are listed in the Supplementary Table 2.

### Minigene assay

The *BCS1L* exon 1, intron 1-2, exon 2, intron 2-3, and exon 3 fragments were cloned into pcDNA 3.1. The pcDNA3.1 plasmid was co-transfected with PCMV-USP39 or PCMV-NC along with USP39 small interfering RNA (siRNA) or control RNA into HEK293T cells for 48 h, and then the RNA was extracted and reverse-transcribed into cDNA as previously described. Semi-quantitative RT-PCR was used to detect alternatively spliced isoforms, and the PCR products were examined by DNA agarose gel electrophoresis.

### Immunoblotting

Cultured cells were lysed on ice with cell lysis buffer for Western blotting and immunoprecipitation (IP) (Beyotime, P0013J), and the protein concentration of the supernatant was measured using the bicinchoninic acid protein assay (Beyotime, P0010). Protein samples were separated by SDS–PAGE and electro-transferred to a PVDF membrane at 200 mA. The PVDF membrane was blocked by 5% skimmed milk for 1 h and then incubated overnight with primary antibodies at 4 °C. All primary antibodies were diluted 1:1000 except for anti-alpha tubulin, which was diluted 1:5000. Proteins of interest were detected with horseradish peroxidase-conjugated secondary antibodies (1:10,000 dilution, Jackson ImmunoResearch) and the ECL system (GE Healthcare). All antibody information is listed in Supplementary Table 1.

### Transmission electron microscopy

A2780 cells were fixed in 2.5% glutaraldehyde. After washing with 0.1 M PBS (pH 7.4) three times for 15 min each, the samples were post-fixed with 1% O_S_O_4_ in 0.1 M PBS (pH 7.4) for 2 h at room temperature. This was followed by one wash with 100% ethanol and two washes with acetone for 15 min each. The samples were embedded in plastic, sectioned, and stained with uranyl acetate and lead citrate. Thin sections were imaged on a Hitachi HT7800 transmission electron microscope. Mitochondrial cristae width was measured using the Image J Multimeasure plug-in.

### Immunohistochemistry staining

Immunohistochemistry staining of 4% formalin-fixed FT and SOC tissues was performed using an immunohistochemistry staining kit (ZSGB-BIO). After paraffin embedding, the sections were deparaffinized with xylene and rehydrated with ethanol. EDTA buffer was used for antigen retrieval by heating in a microwave. Tissue slides were blocked with 5% BSA followed by incubating with primary anti-BCS1L antibodies (1:200 dilution) overnight at 4 °C. The staining score was determined by two pathologists in a blinded manner according to the intensity and extent of staining. The intensity of staining was scored as 0 (negative), 1 (weak), 2 (moderate), or 3 (strong). The extent of staining was based on the percentage of positive BCS1L cells: 1 (0–25%), 2 (26–50%), 3 (51–75%), and 4 (76–100%). The percentage score was multiplied by the staining intensity score to generate the immunohistochemistry staining score. The score of each slide was determined by the average of five fields. The primary antibodies are listed in Supplementary Table 1.

### Immunofluorescence

A2780 cells were grown on coverslips in six-well plates, washed with PBS, fixed with 4% paraformaldehyde for 30 min at room temperature, and permeabilized with 0.5% Triton X-100 in PBS for 15 min and incubated with 5% BSA in PBS for 1 h at room temperature. Primary antibodies (1:500 dilution) against BCS1L and COX4 were added to the blocking buffer at 4 °C overnight. The next day, after rinsing three times in TBST, Alexa Fluor 488- and 594-conjugated secondary antibodies (1:500 dilution, Thermo Fisher Scientific) were added for 1 h at room temperature in a blocking buffer. DAPI (Sigma) was used to stain cell nuclei. Primary antibodies were detected by incubating with secondary antibodies for 1 h at room temperature. The confocal imaging was acquired on an Andor Revolution confocal microscope system (Dragonfly 200) using a ×63 oil objective. For confocal imaging of live cells, 5 × 10^5^ A2780 cells and 3 × 10^5^ HeLa cells were seeded into a 20 mm glass-bottom cell culture dish (NEST). After transfection as indicated, cells were incubated with Mito-Tracker (Beyotime) at 1:50,000 dilution and Hoechst (Solarbio) at 1:100 dilution for 20 min. The glass-bottom cell culture dishes were imaged using a DeltaVision OMX Flex (GE Lifescience). Quantification of the area of colocalization between BCS1L and COX4 was obtained by calculating Manders’ colocalization coefficient and Pearson’s correlation coefficient using the ImageJ Color2 plug-in. Fluorescence-integrated density measurements were made in ImageJ software. The primary antibodies used for immunofluorescence are shown in Supplementary Table 1.

### Co-immunoprecipitation

A total of 1 × 10^7^ A2780 cells with exogenously expressed 3× FLAG-tagged USP39 were harvested and lysed on ice for 30 min with Western and IP Cell Lysis Buffer (Beyotime). The supernatant was transferred to a new 1.5 mL tube and incubated with 5 µg anti-FLAG antibodies (Sigma) for 1 h and then incubated with protein A/G magnetic beads (MCE, HY-K0202) for 2 h. The beads were washed with Western and IP Cell lysis Buffer and boiled for 10 min in 1× SDS–PAGE loading buffer. The samples were confirmed by Western blotting using anti-BCS1L antibodies. The Coomassie blue-stained gel samples were analyzed by LC–MS.

### Blue Native (BN)-PAGE and silver staining

Mitochondria isolated from 1 × 10^7^ A2780 cells were suspended in solubilization buffer (1.5 M aminocaproic acid, 50 mM Bis–Tris/HCl, pH 7.0) with digitonin (0.12 mg digitonin per mg mitochondria) on ice for 30 min. Following solubilization, the samples were centrifuged at 14,000×*g* for 30 min at 4 °C to remove insoluble debris. The protein concentration was determined, and 50 µg protein in non-denaturing Gel Protein Sample Loading Buffer (Beyotime, P0016) was loaded onto a 4–12% precast gradient gel (YEASON, 36249ES10). The gel was run for about 1 h at 140 V in a cold room. After electrophoresis, the complexes were transferred to the PVDF membrane and incubated with primary antibodies. Silver staining was performed with a Fast Silver Stain Kit (Beyotime, P0017S) following the manufacturer’s instructions.

### RNA pull-down assay

*BCS1L* pre-mRNA with a T7 promoter was cloned from human placenta genomic DNA and then transcribed into DNA fragments using an RNAMAX-T7 in vitro transcription kit (Vazyme). The fragments were labeled with biotin using the Pierce RNA 3′ End Desthiobiotinylation Kit (Thermo Fisher Scientific), and RNA pull-down was conducted using the Magnetic RNA Protein Pull-Down Kit (Thermo Fisher Scientific) following the manufacturer’s protocol. Poly(A)_25_ RNA served as the negative control, while the positive control was the 3′ untranslated region of the androgen receptor RNA. The proteins were detected by western blot analysis and mass spectrometry.

### RNA immunoprecipitation with sequencing (RIP-seq)

A2780 cells with ectopically expressed FLAG-tagged USP39 were cross-linked with 0.3% formaldehyde, and nuclei extracted from the cross-linked cells were lysed in RIP lysis buffer with RNase and protease inhibitor. The DNA was sheared into 500–1000 bp fragments by sonication and was incubated with magnetic beads coated with anti-FLAG antibody (Cell Signaling Technology). RNA was extracted with phenol/chloroform/isoamyl alcohol (125:24:1 mixture) and digested by DNase I. Both input and RIP samples were prepared for next-generation sequencing by Ribobio Biotechnology Company. The sequencing results were aligned with the inputs, and the clean reads were re-mapped to an annotated human genome (hg19). The RIP-seq data generated in this study have been deposited in the NCBI GEO database under the accession number GSE157401.

### ScRNA-seq data processing

The original scRNA-seq data were derived from a GSA-Human under accession code PRJCA005422. For visualization, the dimensionality of cancer cells was further reduced using the uniform manifold approximation and projection (UMAP) with the Seurat function Run-UMAP. Cells from the epithelial cluster were extracted and reclustered for 9 subclusters by dimension 8, setting the clustering resolution to 0.3. The differentially expressed genes of each cell subcluster were identified by the FindAllMarkers function of Seurat (V.4.3.0). Pathway enrichment in ascites and primary tumor was performed with the irGSEA package (V.1.1.3) (https://github.com/chuiqin/irGSEA). The predefined sets of genes involved in OXPHOS in the MSigDB database were used for analysis. The primary subunits of the five mitochondrial complexes were listed in Supplementary Table S4.

### Statistics

The gene expression profiles were analyzed based on the TCGA and GTEx databases. Differential gene expression was calculated with read counts after normalization, and the functional enrichment analysis of differentially expressed genes was conducted using GO and GSEA. All statistical analyses were conducted using GraphPad Prism 8 and R (version 3.6.3). Differences between control and treatment conditions were calculated using Student’s unpaired *t*-test, and one-way ANOVA with Dunnett’s post-test was used to compare test conditions to controls. Pearson’s correlation coefficient and Manders’ coefficient were used to determine the colocalization. All results are shown as the means ± SD of three independent experiments. *P*-values < 0.05 were considered statistically significant. All experimental results were repeated at least three times with independent samples. Statistical details for each experiment are indicated in the figure legends.

### Ethics approval and consent to participate

All experiments were performed in accordance with the relevant guidelines and regulations. The animal experiment was approved by the Shandong University Animal Ethics Research Board (SDULCLL2019-2-08). The ovarian cancer tissues were collected from primary ovarian cancer patients at Qilu Hospital of Shandong University. Informed consent was obtained from all patients.

## Supplementary information

Supplementary Figures and legends

Supplementary Tables

## Data Availability

All data associated with this study are available in the main text or the supplementary materials. Further information and requests for resources and reagents should be directed to and will be fulfilled by the lead contact, Zhaojian Liu (liujian9782@sdu.edu.cn).
