# Supplementary material for: Splice-switching of the oncogenic BCS1L isoform suppresses ovarian cancer progression by disrupting mitochondrial function
Source: Cell Death Dis. 2026 Mar 3;17(1):293. doi: 10.1038/s41419-026-08495-6 (PMC13039997; doi:10.1038/s41419-026-08495-6)
Supplement: Supplementary file 1 — Supplementary Figures and legends [file 41419_2026_8495_MOESM1_ESM.pdf]

Supplemental Information

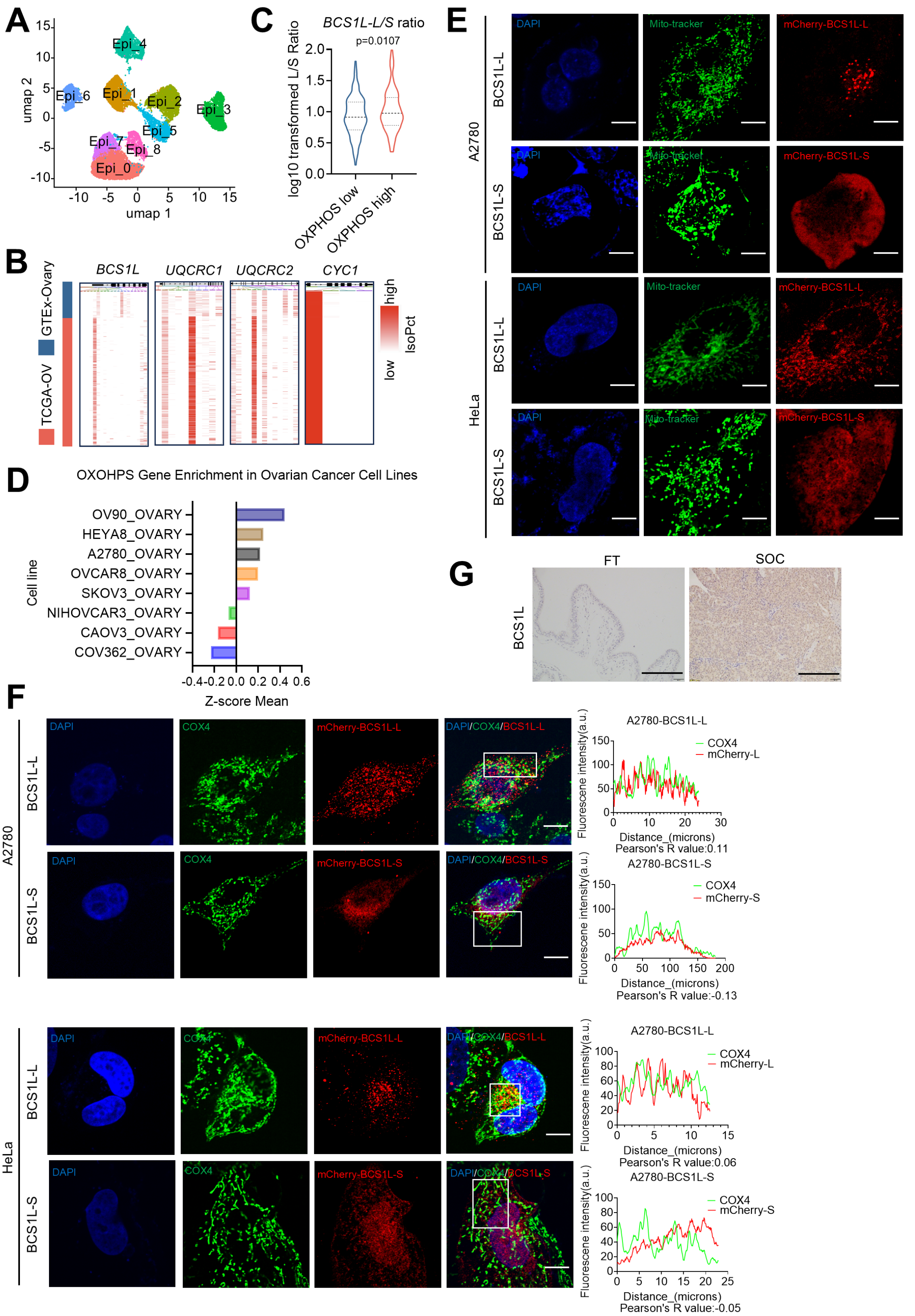

Figure S1. *BCS1L-L* or *BCS1L-S* expression difference and subcellular localization.

(A) UMAP plot illustrated nine epithelial subclusters after dimension reduction colored by subclusters. **(B)** The main isoforms expression of mitochondrial complex III subunit BCS1L,

UQCRC1, UQCRC2 and CYC1 in GTEx-ovary and TCGA-OV datasets. **(C)** The BCS1L-L/BCS1L-S value transformed to log10 in OXPHOS low or OXPHOS high group from TCGA-OV, p = 0.0107. **(D)** The 184 oxidative phosphorylation–associated genes enrichment in ovarian cancer cell lines (OV90, HEYA8, A2780, OVCAR8, SKOV3, NIHOVCAR3, CAOV3 and COV362) based on CCLE datasets. The OXPHOS genes expression was evaluated by Z-score mean value. **(E)** Immunofluorescence image of A2780 and HeLa live cells transfected with pUltra-hot-BCS1L-L and pUltra-hot-BCS1L-S plasmids respectively for 72 h showing localization of BCS1L-L and BCS1L-S (red). Mito-tracker (green) indicated the mitochondria localization and DAPI (blue) was used to visualize nuclei. Scale bars, 10 µm. **(F)** Colocalization of BCS1L-L or BCS1L-S protein labeled by mCherry (red) with mitochondria marked by COX4 (green) in control and pUltra-hot-BCS1L-L or pUltra-hot-BCS1L-S plasmids transfection for 72 h in A2780 and HeLa. DAPI (blue) was used to visualize nuclei. The quantification of co-localization was performed with image J Coloc 2 and Plot Profile. Scale bars, 10 µm. **(G)** Representative images of IHC staining of BCS1L in FT and SOC tissues. Scale bars, 200 µm.

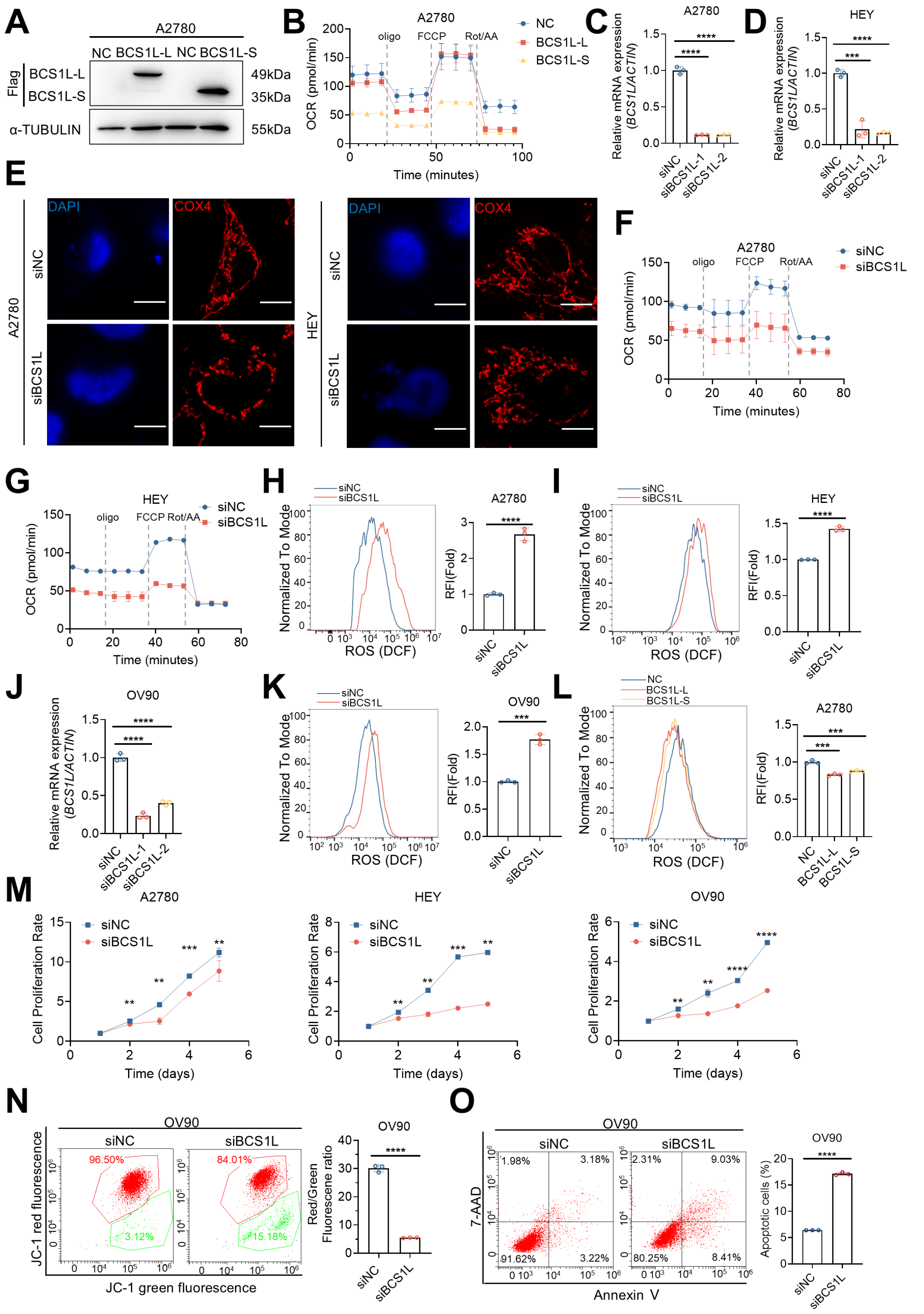

Figure S2. BCS1L knockdown impairs mitochondrial homeostasis in ovarian cancer cells. **(A)** The protein level of BCS1L-L and BCS1L-S in A2780 with or without Flag-BCS1L-L or Flag-BCS1L-S overexpression vectors. **(B)** The OCR curve of A2780 with BCS1L-L or BCS1L-S overexpression and control for 72 h measured by Agilent Seahorse XFe24 after the oligomycin, FCCP and rotenone/antimycin injections. **(C)** ROS level in A2780 cell transfected

with control and BCS1L-L or BCS1L-S vectors for 48 h was detected by ROS probe DCFH-DA on a flow cytometer. **(D-E)** The efficiency of a siRNA suit silencing BCS1L in A2780 and HEY cell lines. **(F)** Confocal image of COX4 (red) and DAPI (blue) in A2780 and HEY cells with BCS1L knockdown and control showing mitochondria morphology. DAPI was used to visualize nuclei. Scale bars, 10 μm. **(G-H)** The OCR curve of A2780 and HEY with BCS1L knockdown and control measured by Agilent Seahorse XFe96 after the oligomycin, FCCP and rotenone/antimycin injections. **(I)** The efficiency of a siRNA suit silencing BCS1L in OV90 cell line. **(J-L)** ROS level was detected by flow cytometry in A2780, HEY and OV90 cells with BCS1L depletion for 48 h. **(M)** Mitochondrial membrane potential analyzed by flow cytometry using JC-1 staining in OV90 cells with BCS1L knockdown for 72 h. **(N)** Cell proliferation was measured by the MTT assay in ovarian cancer cells (A2780, HEY, OV90) with BCS1L knockdown compared to corresponding controls (n = 3 biologically independent experiments for the cell proliferation assay). Absorbance at 570 nm at each time point was compared to the initial value on the first day. **(O)** Apoptotic cells analyzed by flow cytometry using Annexin V/7-AAD staining in OV90 cells with BCS1L knockdown for 72 h. All results are presented as the mean ± SD. The p-value was obtained by unpaired two-sided Student's t-test. **$p < 0.01$, ***$p < 0.001$, ****$p < 0.0001$.

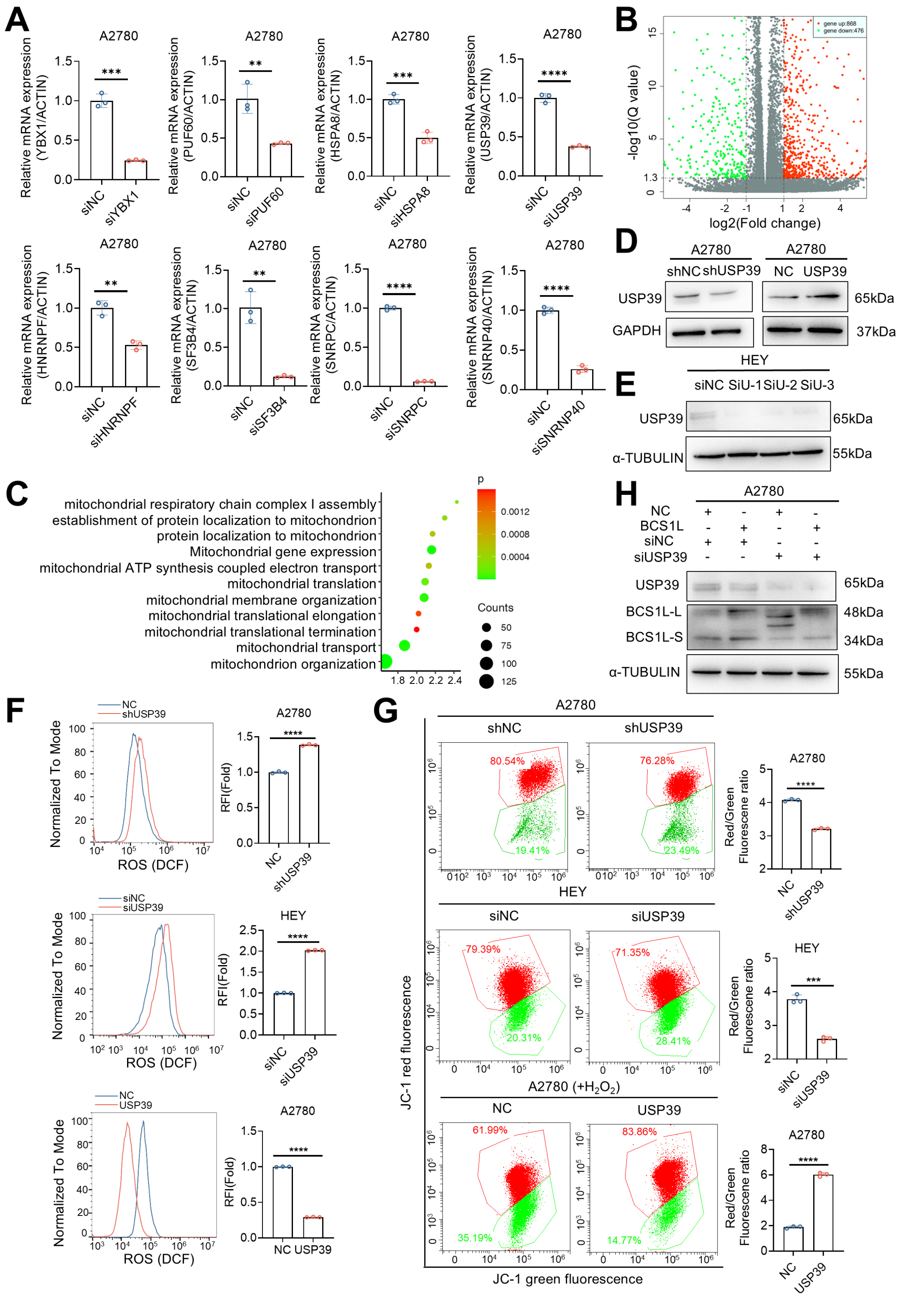

Figure S3. USP39-BCS1L axis mediates mitochondrial oxidative phosphorylation in ovarian cancer cells.

(A) The knockdown efficiency of all the siRNA (siYBX1, siPUF60, siHSPA8, siUSP39, siHNRNPF, siSF3B4, siSNRPC, siSNRNP40) compared with control in A2780 cells was demonstrated by qPCR. **(B)** Volcano plot of differentially expressed genes (DEGs) in A2780 cells with USP39 knockdown. The cutoff was set as q-value < 0.05 and |fold change (FC)| >1.

868 genes were upregulated, and 476 genes were downregulated. **(C)** GO biological process enrichment analysis according to DEGs in the RNA-seq data from A2780 cells after USP39 stable knockdown. **(D)** USP39 protein level in stably USP39-depleted A2780 cell lines and stably USP39-overexpressed A2780 cell lines. **(E)** The efficiency of a siRNA suit silencing USP39 was verified with western blot. **(F)** ROS level of A2780 and HEY with USP39 depletion and A2780 with USP39 overexpression were detected by ROS probe DCFH-DA on a flow cytometer. RFI, relative fluorescence intensity. **(G)** Flow cytometry analysis of A2780 and HEY with USP39 knockdown and A2780 overexpressing USP39 stained with JC-1, indicating the level of mitochondrial membrane potential. Three biological replicates were conducted in all functional experiments. **(H)** Western blot analysis of BCS1L and USP39 protein expression in A2780 cells. The p values were obtained by a two-tailed unpaired Student's t-test **(A, F, G, H, I)**, $*p < 0.05$, $**p < 0.01$, $***p < 0.001$, $****p < 0.0001$. Data are mean ± SD.

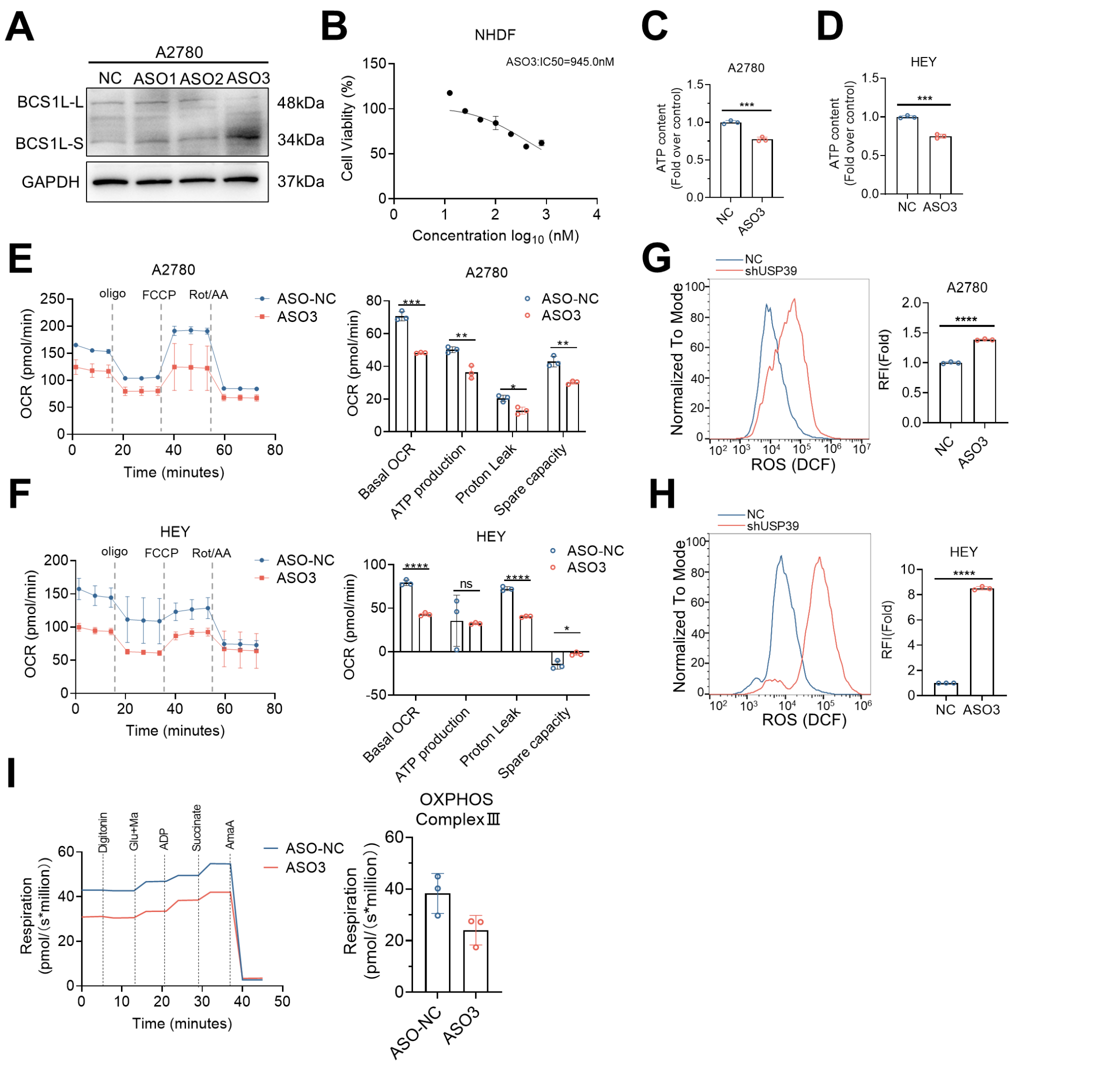

Figure S4. Antisense oligonucleotides targeting BCS1L induce mitochondrial dysfunction specifically in ovarian cancer cells.

(A) The BCS1L isoform expression in A2780 cells transfected with three ASOs with 100nM for 48h detected by western blot. **(B)** The IC50 of ASO3 was measured in Normal Human Dermal Fibroblasts (NHDF) at 945.0 nM. **(C-D)** The total ATP content of whole cells was detected in A780 cells with 200 nM ASO3 and HEY with 100 nM ASO3 for 48 h compared with the corresponding control. **(E-F)** Mitochondrial basal OCR, Maximal OCR, Proton leak and spare capacity were analyzed by Agilent Seahorse XFe96 after oligomycin, FCCP, and rotenone/antimycin injection in A2780 and HEY cells treated with ASO3 100 nM and 50 nM for 24 h. **(G-H)** ROS level was analyzed by flow cytometry followed by DCFH-DA staining in A2780 treated with 200 nM and HEY treated with 100 nM for 48 h. **(I)** ETC Complex III activities in A2780 cells transfected with 100 nM was evaluated by substrate-uncoupler-inhibitor-titration (SUIT) high-resolution Oxygraph-2k respirometer, $p = 0.0631$. The p values were obtained by a two-tailed unpaired Student's t-test **(C, D, E, F, G, H, I)**, *$p < 0.05$, **$p < 0.01$, ***$p < 0.001$, ****$p < 0.0001$. ns: not significant. Data are mean ± SD.
